# Identification of immunogenic cell death-related gene classification patterns and immune infiltration characterization in ischemic stroke based on machine learning

**DOI:** 10.3389/fncel.2022.1094500

**Published:** 2022-12-19

**Authors:** Jiayang Cai, Zhang Ye, Yuanyuan Hu, Ji’an Yang, Liquan Wu, Fanen Yuan, Li Zhang, Qianxue Chen, Shenqi Zhang

**Affiliations:** ^1^Department of Neurosurgery, Renmin Hospital of Wuhan University, Wuhan, Hubei, China; ^2^Central Laboratory, Renmin Hospital of Wuhan University, Wuhan, Hubei, China; ^3^Department of Ophthalmology, Tongji Hospital, Tongji Medical College, Huazhong University of Science and Technology, Wuhan, Hubei, China; ^4^Department of Anesthesiology, Renmin Hospital of Wuhan University, Wuhan, Hubei, China

**Keywords:** immunogenic cell death, immune infiltration, ischemic stroke, machine learning, immunotherapy

## Abstract

Ischemic stroke (IS) accounts for more than 80% of strokes and is one of the leading causes of death and disability in the world. Due to the narrow time window for treatment and the frequent occurrence of severe bleeding, patients benefit less from early intravenous thrombolytic drug therapy. Therefore, there is an urgent need to explore the molecular mechanisms poststroke to drive the development of new therapeutic approaches. Immunogenic cell death (ICD) is a type of regulatory cell death (RCD) that is sufficient to activate the adaptive immune response of immunocompetent hosts. Although there is growing evidence that ICD regulation of immune responses and immune responses plays an important role in the development of IS, the role of ICD in the pathogenesis of IS has rarely been explored. In this study, we systematically evaluated ICD-related genes in IS. The expression profiles of ICD-related genes in IS and normal control samples were systematically explored. We conducted consensus clustering, immune infiltration analysis, and functional enrichment analysis of IS samples using ICD differentially expressed genes. The results showed that IS patients could be classified into two clusters and that the immune infiltration profile was altered in different clusters. In addition, we performed machine learning to screen nine signature genes that can be used to predict the occurrence of disease. We also constructed nomogram models based on the nine risk genes (CASP1, CASP8, ENTPD1, FOXP3, HSP90AA1, IFNA1, IL1R1, MYD88, and NT5E) and explored the immune infiltration correlation, gene-miRNA, and gene-TF regulatory network of the nine risk genes. Our study may provide a valuable reference for further elucidation of the pathogenesis of IS and provide directions for drug screening, personalized therapy, and immunotherapy for IS.

## Introduction

One of the leading causes of death and disability in the world is stroke, with ischemic stroke accounting for more than 80% of cases. With the aging and urbanization of society, the prevalence of unhealthy lifestyles, and exposure to cardiovascular risk factors, the burden of ischemic stroke is rapidly increasing (Hasan et al., 2018). Ischemic stroke (IS) can be a multifactorial disease resulting from the interacting effects of multiple environmental and inherited risk factors (Chehaibi et al., 2016). Despite continued research into IS, early intravenous thrombolytic drug therapy is still a preferred modality, but patients benefit less because of the narrow time window for treatment and the frequent occurrence of severe bleeding (Alexandrov, 2010; de Los Ríos la Rosa et al., 2012). As early diagnosis and treatment of IS face great challenges, there is an urgent need to explore the molecular mechanisms poststroke to drive the development of new therapeutic approaches.

In recent years, several studies have shown that the immune response plays a crucial role in the development of stroke and that neurological function and prognosis can be improved through the regulation of the immune microenvironment of the central nervous system (Javidi and Magnus, 2019; Jayaraj et al., 2019; Krishnan and Lawrence, 2019). Immunogenic cell death (ICD), a type of regulatory cell death (RCD) recommended by the Nomenclature Committee on Cell Death (NCCA), is sufficient to activate the adaptive immune response of immunocompetent hosts (Galluzzi et al., 2018). Damage-associated molecular patterns (DAMPs), including released high mobility group 1 (HMGB1) protein, secreted adenosine triphosphate (ATP) and surface-exposed calreticulin (CRT), are the main immunogenic features of ICD (Krysko et al., 2012). Similarly, cerebral tissue ischemia resulting from IS-induced blockage of cerebral blood flow rapidly causes the release of signaling molecules, including brain-derived antigens, DAMPs, cytokines, and chemokines, from damaged brain tissue into the body circulation (Liu et al., 2021). Therefore, ICD may play an important role in the occurrence and progression of IS. Numerous studies have shown that ICD is significantly involved in the pathogenesis of a variety of diseases, especially in tumor immunity (Kroemer et al., 2022). Recently, a new gene signature in intracranial aneurysms (IAs) has been established through immunogenic cell death-related regulators, which provides a basis for optimizing risk monitoring and clinical decision-making and developing new therapeutic strategies for IA patients (Turhon et al., 2022). However, there is growing evidence that ICDs regulate immune responses and that immune responses serve important roles in the development of IS. However, the role of ICD in the pathogenesis of IS has rarely been explored. Therefore, an in-depth study of the different immune profiles between normal tissues and IS specimens, as well as the different subtypes of IS, will help to elucidate the changes that occur in ICD and its associated genes. Meanwhile, establishing ICD-related signatures will help to improve personalized treatment for patients.

In this study, we systematically evaluated ICD-related genes in IS. We explored the expression profiles of ICD-related genes in IS and normal control samples. We also performed consensus clustering, immunoinfiltration analysis, and functional enrichment analysis of IS samples using ICD differentially expressed genes. In addition, we screened nine risk signature genes using machine learning algorithms that can be used to predict the occurrence of disease and constructed nomogram models. Moreover, the immune infiltration correlation, gene-miRNA, and gene-TF regulatory network of the nine risk genes were explored. Our study could potentially lay the foundation for the development of individualized treatment and immunomodulatory therapeutic regimens for IS.

## Materials and methods

### Datasets

The gene expression omnibus (GEO) database^1^ was used to obtain gene expression profiling datasets of the IS-related peripheral blood samples. Dataset GSE58294 (GPL570 platform), including 23 control samples and 69 IS samples, was used as a training set. Dataset GSE16561 (GPL6883 platform), including 24 control samples and 39 IS samples, was used as a validation set.

### Differentially expressed genes analysis

The R package “Limma” was used to detect differentially expressed genes (DEGs) between normal samples and IS samples. A *p*-value < 0.05 was considered to be statistically significant.

### Immune cell infiltration profile

The CIBERSORT algorithm was used to assess the content of 22 immune cells in each sample. *P* < 0.05 for sample immune infiltration was considered accurate and was used for further data analysis. Then, we compared the fraction of immune cells between different groups through the Wilcoxon test.

### Consensus clustering

Based on DEGs, “ConsensusClusterPlus” was used to perform an unsupervised clustering analysis of IS patients. The cumulative distribution function (CDF) curve, consensus score, and consensus matrix were used to determine the optimal number of subtypes k.

### Gene set variation analysis

The “c2.cp.kegg.symbols” file and the “c5.go.symbols” file were downloaded from the MSigDB database and used to study the changes in the biological signaling pathways. The R packages “GSVA” and “Limma” were used to analyze the altered pathways and biological functions between different clusters.

### Machine learning algorithms

Least absolute shrinkage and selection operator and support Vector machine recursive feature elimination (SVM-RFE) was used to filter important diagnostic variables based on ICD-related DEGs between IS patients and controls. We determined the intersection of the signature genes screened by the two algorithms and generated receiver operating characteristic (ROC) curves separately to determine the predictive value of these signature genes in the training set. The area under the curve (AUC) was calculated using the R package “pROC.” Meanwhile, the predictive power of these signature genes was verified in the validation set. In addition, we also constructed a nomogram with the R package “rms” based on these signature genes.

### Gene ontology and Kyoto encyclopedia of genes and genomes analysis

To explore the differential signaling pathways and potential functions of signature genes, we conducted gene ontology (GO) and Kyoto encyclopedia of genes and genomes (KEGG) enrichment analyses of these genes by using the R package “clusterProfiler,” and a *q*-value < 0.05 was considered significant.

### Correlation of immune-infiltrating cells with signature genes

The correlation coefficient between the expression of ICD-related genes and the immune-infiltrating cells was calculated to explore the relationship between immune-infiltrating cells and signature genes by using Spearman’s rank correlation analysis. The R package “ggplot” was used to plot the Lollipop plots.

### Construction of regulatory networks

NetworkAnalyst^2^ was used to construct the miRNA diagnostic biomarker and transcription factor (TF)-diagnostic biomarker regulatory networks based on signature genes (Xia et al., 2015).

### Statistical analysis

Bioinformatics analyses and R packages were all conducted by R software (version 4.2.0). The means between two groups of normally distributed variables were compared using unpaired Student’s *t*-tests. Data that were not normally distributed were compared by the Wilcoxon test. *P < 0.05, **P < 0.01, and ***P < 0.001 were regarded as significant.

## Results

### Expression landscape of ICD-related genes

**FIGURE 1:**
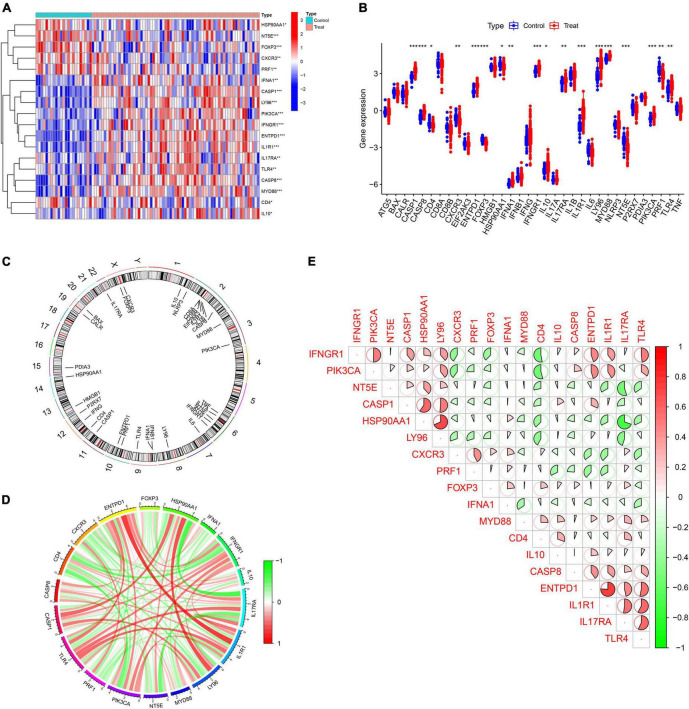
Expression profile of immunogenic cell death (ICD)-related genes in IS. **(A,B)** Heatmap and boxplots showing the expression of 18 differentially expressed ICD-related genes. **(C)** The relative positions of the 18 differentially expressed ICD-related genes on the chromosome. **(D)** The correlation circle plot shows the degree of correlation of the 18 differentially expressed ICD-related genes. **(E)** Correlation heatmap showing correlation coefficients for 18 differentially expressed ICD-related genes. Red and green represent positive and negative correlations, respectively. The correlation coefficient is displayed as the area of the pie chart. *P < 0.05, **P < 0.01, ***P < 0.001.

In a previous large-scale meta-analysis, Garg et al. (2016) summarized 34 ICD-related genes. We explored the expression patterns of 34 ICD genes in IS samples and healthy control samples, and the results showed that most ICD genes were highly expressed in IS samples, including CASP1, CASP8, ENTPD1, IFNA1, IFNGR1, IL10, IL17RA, IL1R1, LY96, MYD88, PIK3CA, and TLR4, while CD4, CXCR3, FOXP3, HSP90AA1, NT5E, and PRF1 were expressed at low levels in IS samples (Figures 1A,B). Meanwhile, the chromosome positions of the 34 ICD genes were visualized (Figure 1C). Next, a correlation analysis of these differentially expressed ICD genes was performed to explore the interactions between them (Figures 1D,E).

### Identification of ICD clusters based on ICD-related DEGs

**FIGURE 2:**
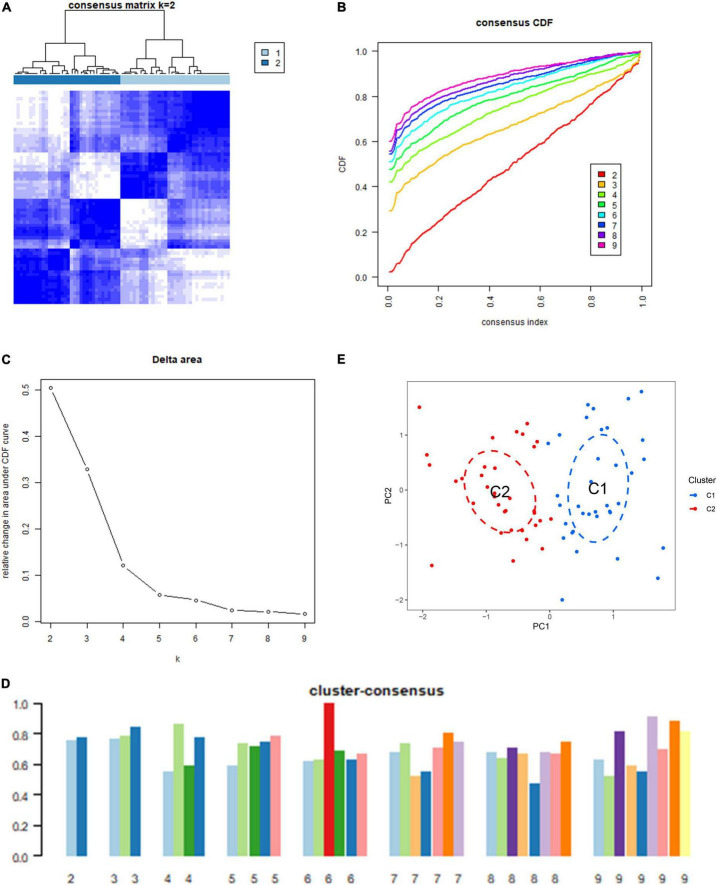
Identification of the immunogenic cell death (ICD) clusters based on ICD-related differentially expressed genes (DEGs). **(A)** Consensus clustering matrix when *k* is 2. **(B)** Representative cumulative distribution function (CDF) curve. **(C)** Representative CDF delta area curve. **(D)** Consensus clustering score when *k* is 2–9. **(E)** Visualization of the distribution of the two clusters by principal component analysis (PCA).

Based on 18 ICD-related DEGs, we divided IS samples into two clusters (C1 and C2). We set the value of *k* to 1–9 and found that the consensus index of the CDF curve fluctuates the least and that the consensus score is relatively large when *k* = 2 (Figures 2A–D). Moreover, principal component analysis (PCA) results showed that the 18 DEGs can completely distinguish between the two clusters (Figure 2E).

### Identification of immune microenvironment and biological function characteristics in different ICD clusters

**FIGURE 3:**
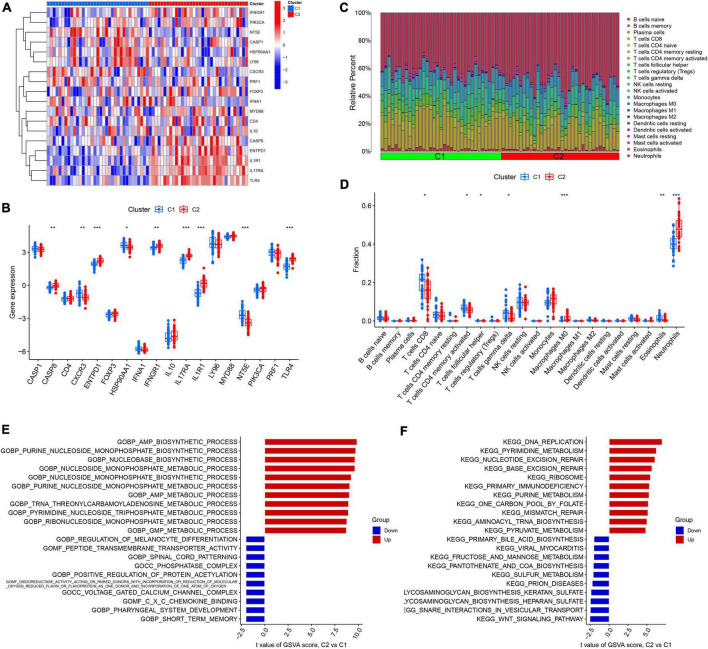
Identification of immune infiltration and biological function characteristics in different immunogenic cell death (ICD) clusters. **(A)** Heatmap showing the expression profile of 18 ICD-related differentially expressed genes (DEGs) between two ICD clusters. **(B)** Boxplots showing differences in the expression of 18 ICD-related DEGs between the two ICD clusters. **(C)** Relative abundance of 22 infiltrating immune cells between the two ICD clusters. **(D)** Boxplots showing differences in immune infiltration between the two ICD clusters. **(E)** Gene set variation analysis (GSVA) results of gene ontology (GO) gene sets between two ICD clusters were plotted in a bar plot. **(F)** GSVA results of Kyoto encyclopedia of genes and genomes (KEGG) gene sets between two ICD clusters were plotted in a bar plot. *P < 0.05, **P < 0.01, ***P < 0.001.

We analyzed the difference in 18 DEGs between different ICD clusters and found that CASP8, ENTPD1, IFNGR1, IL17RA, IL1R1, and TLR4 were upregulated in Cluster 2, while CXCR3 and NT53 were upregulated in Cluster 1 (Figures 3A,B). To further explore the differences in the immune microenvironment features between the different ICD clusters, the differences in infiltrating immune cells and their immune functions were analyzed. Our results showed that Cluster 2 had relatively low levels of CD8+ T cells, follicular helper T cells, activated memory CD4+ T cells, eosinophils, and gamma delta T cells and relatively high levels of M0 macrophages and neutrophils (Figures 3C,D). Next, we conducted GSVA based on GO and KEGG gene sets. The GO results showed that AMP metabolic processes and GMP metabolic processes were upregulated in Cluster 2, while positive regulation of protein acetylation and CXC chemokine binding were downregulated in Cluster 2 (Figure 3E). The KEGG results showed that DNA replication and primary immunodeficiency were upregulated in Cluster 2, while the WNT signaling pathway, pantothenate, and COA biosynthesis were downregulated in Cluster 2 (Figure 3F).

### Identification of the gene clusters based on the DEGs of ICD clusters

**FIGURE 4:**
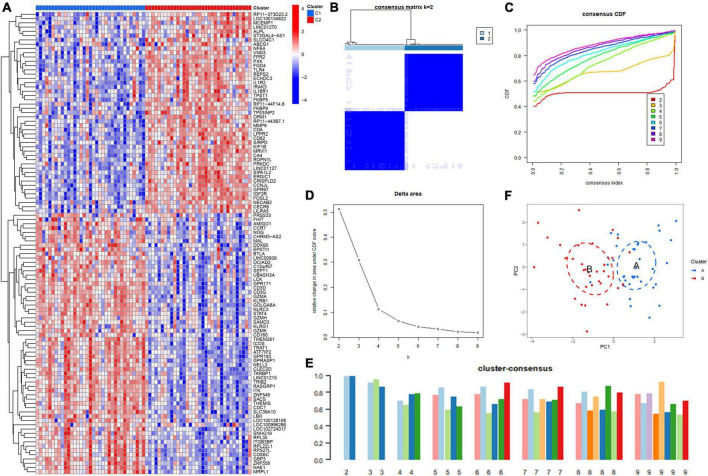
Identification of the gene clusters based on the differentially expressed genes (DEGs) of immunogenic cell death (ICD) clusters. **(A)** Heatmap showing the DEGs between ICD clusters. **(B)** Consensus clustering based on 108 DEGs and the consensus clustering matrix when *k* is 2. **(C)** Representative cumulative distribution function (CDF) curve. **(D)** Representative CDF delta area curve. **(E)** Consensus clustering score when *k* is 2–9. **(F)** Visualization of the distribution of the two gene clusters by principal component analysis (PCA).

To further validate the ICD clusters, we screened for DEGs between Cluster 1 and Cluster 2 and found 108 DEGs in total (Figure 4A). Based on the 108 DEGs, we divided the IS patients into different genomic subtypes (Cluster A and Cluster B) by using consensus clustering. We observed that the optimal grouping was obtained when *k* = 2, and the consensus score had a maximum value (Figures 4B–E). Moreover, the PCA showed that the 108 DEGs can completely distinguish between the two clusters (Figure 4F). These results suggested that two different clusters exist in IS patients.

### Identification of immune microenvironment and biological function characteristics in different gene clusters

**FIGURE 5:**
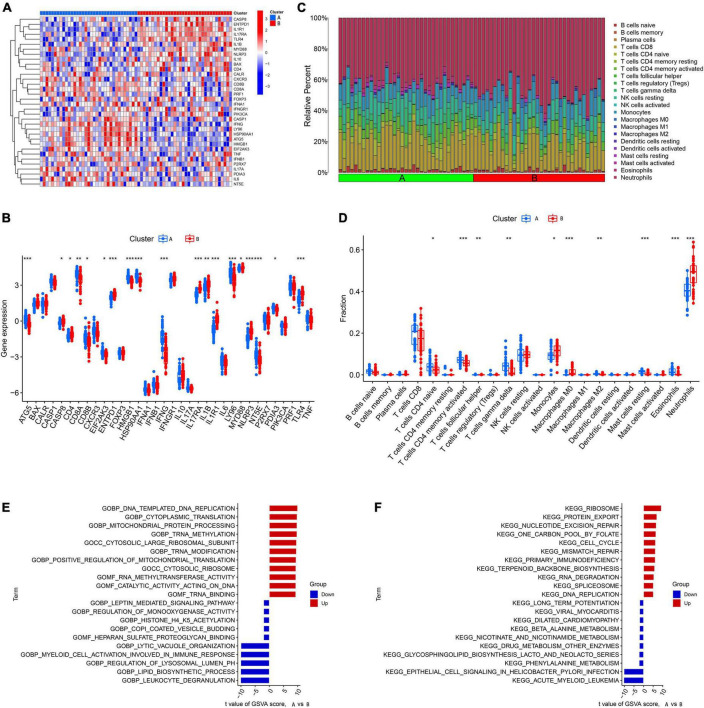
Identification of immune infiltration and biological function characteristics in different gene clusters. **(A)** Heatmap showing the expression profile of 34 immunogenic cell death (ICD)-related differentially expressed genes (DEGs) between two gene clusters. **(B)** Boxplots showing differences in the expression of 34 ICD-related DEGs between the two gene clusters. **(C)** Relative abundance of 22 infiltrating immune cells between the two gene clusters. **(D)** Boxplots showing differences in immune infiltration between the two gene clusters. **(E)** Gene set variation analysis (GSVA) results of gene ontology (GO) gene sets between two gene clusters were plotted in a bar plot. **(F)** GSVA results of Kyoto encyclopedia of genes and genomes (KEGG) gene sets between two gene clusters were plotted in a bar plot. *P < 0.05, **P < 0.01, ***P < 0.001.

We first explored the different expression profiles of 34 ICD-related genes between Cluster A and Cluster B (Figures 5A,B). The results of infiltrating immune cells showed that there were more differential immune cells between different genetic groupings. Cluster B was characterized by low levels of naive CD4+ T cells, follicular helper T cells, activated memory CD4+ T cells, gamma delta T cells, M2 macrophages, eosinophils, and resting mast cells and high levels of monocytes, M0 macrophages, and neutrophils (Figures 5C,D). These results suggest that gene clusters may be able to characterize IS patients better than ICD clusters. Next, we conducted GSVA between different gene clusters. We found that pathways involved in mitochondrial protein processing, cytoplasmic translation, primary immunodeficiency, and the cell cycle were upregulated in Cluster B, while pathways involved in the immune response, such as myeloid activation and leukocyte degranulation, were upregulated in Cluster B (Figures 5E,F).

### Construction and validation of the LASSO model and SVM model

**FIGURE 6:**
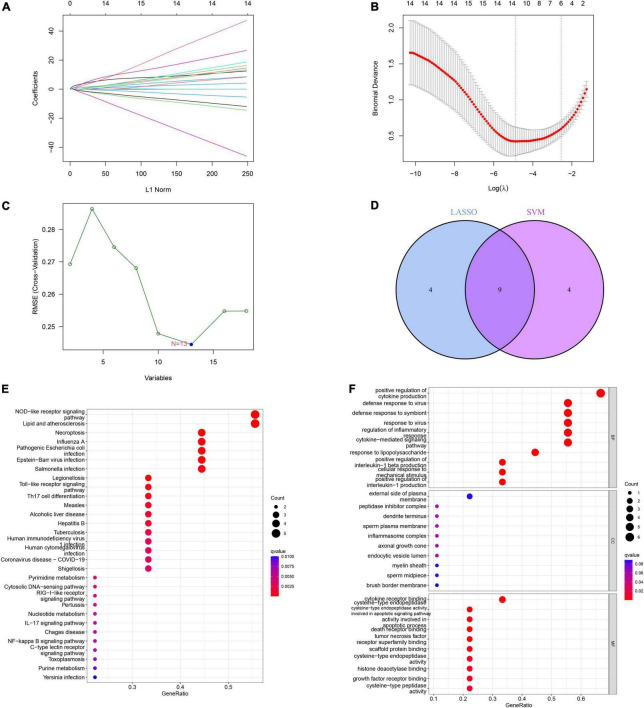
Construction and validation of the least absolute shrinkage and selection operator (LASSO) model and support vector machine (SVM) model. **(A)** The LASSO coefficient profiles of differentially expressed genes (DEGs) in ischemic stroke (IS) samples. **(B)** Partial likelihood deviance for the LASSO coefficient profiles. Thirteen genes were selected at the value (lambda.min). **(C)** The root mean square error (RMSE) was calculated from 15-fold CV and verified the results of support Vector machine recursive feature elimination (SVM-RFE). The highlighted point indicates the lowest error rate, and the corresponding genes at this point are the best signature genes selected by SVM. **(D)** Venn diagram demonstrating nine immunogenic cell death (ICD)-related signature genes shared by the LASSO and SVM algorithms. **(E)** Bubble plot of Kyoto encyclopedia of genes and genomes (KEGG) analysis results based on the nine signature genes. **(F)** Bubble plot of gene ontology (GO) analysis results based on the nine signature genes.

We established a LASSO and SVM model to select candidate ICD genes from the 18 ICD-related DEGs to predict the occurrence of IS. The LASSO model results showed that 13 genes were related to the occurrence of IS (Figures 6A,B). Meanwhile, the feature vectors generated by SVM were removed using a support vector machine (SVM) to find the best variables and identify 13 ICD variable genes (Figure 6C). Finally, we took the intersection of the genes obtained from the two machine learning models and left nine signature genes, including CASP1, CASP8, ENTPD1, FOXP3, HSP90AA1, IFNA1, IL1R1, MYD88, and NT5E, for subsequent analysis (Figure 6D). We conducted GO and KEGG enrichment analyses based on the nine signature genes. The KEGG results showed that these genes were enriched in necroptosis, Th17 cell differentiation, the IL-17 signaling pathway, the NF-kappa B signaling pathway, and other pathways (Figure 6E). The GO results showed that these genes are mainly involved in the positive regulation of cytokine production, regulation of inflammatory response, positive regulation of interleukin-1 beta production, tumor necrosis factor receptor superfamily binding, and apoptotic signaling pathway (Figure 6F).

**FIGURE 7:**
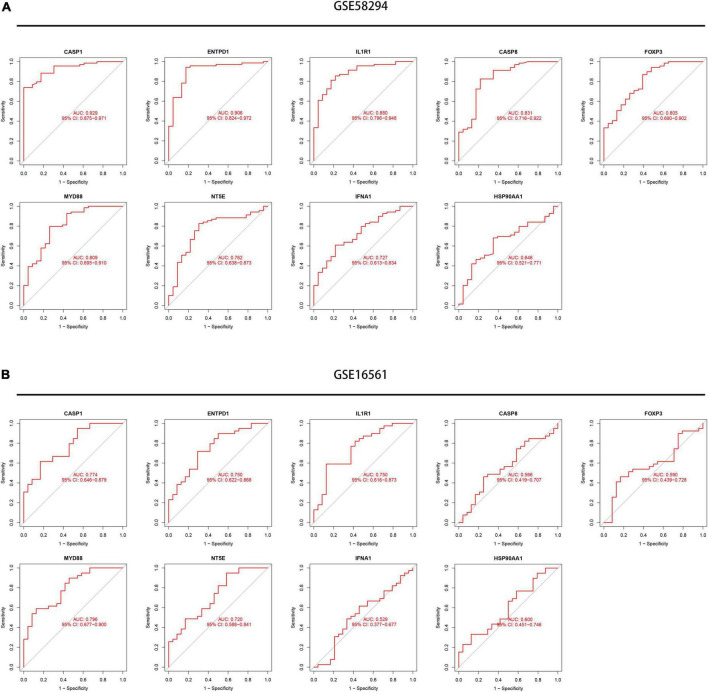
Exploration of the diagnostic value of the nine signature genes. **(A)** Receiver operating characteristic (ROC) curves showing the diagnostic value of nine signature genes in the GSE58294 dataset. **(B)** ROC curves showing the diagnostic value of nine signature genes verified by the GSE16561 dataset.

Next, we conducted an ROC analysis to evaluate the accuracy of each diagnostic gene, and the AUC value of the ROC curve was also calculated. Our results showed that all nine genes had relatively high predictive values in the training set (GSE58294), especially CASP1 and ENTPD1 (Figure 7A). Meanwhile, we performed validation in another dataset and obtained similar results (Figure 7B). Meanwhile, we explored the expression levels of nine signature genes in the GSE16561 dataset (Supplementary Figures 1A,B).

### Construction of the nomogram model

**FIGURE 8:**
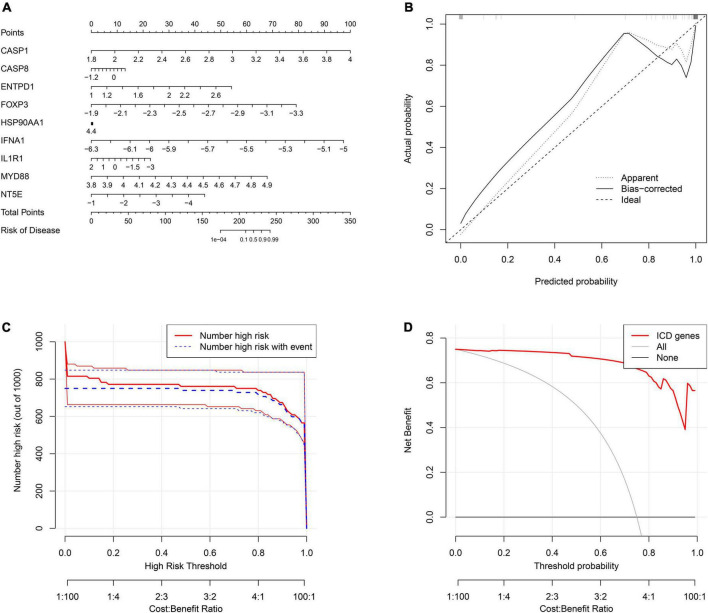
Construction of the nomogram model. **(A)** The ordinary nomogram for the joint diagnosis of ischemic stroke (IS) based on CASP1, CASP8, ENTPD1, FOXP3, HSP90AA1, IFNA1, IL1R1, MYD88, and NT5E. **(B)** Calibration curve for nomogram validation. **(C)** Clinical impact of the nomogram model as assessed by the clinical impact curve. **(D)** Decision curve analysis based on the nomogram model.

To better predict the risk of patient incidence, we constructed a nomogram based on the nine diagnostic genes (Figure 8A). Each gene in the nomogram is projected upward to a point, and the sum of the scores of the three variables is transformed into an individual’s disease risk, in which a high overall score corresponds to a higher disease risk. The results of the calibration curve indicated that the predictive ability of the nomogram model was accurate (Figure 8B). The clinical impact curve also showed the significant predictive power of the nomogram model (Figure 8C). In addition, the red line in the decision curve analysis (DCA) curve from 0 to 1 is consistently higher than the gray and black lines, suggesting that the decision based on the nomogram model may benefit pediatric asthma patients (Figure 8D).

### Immune infiltration correlation analysis and construction of regulatory networks

**FIGURE 9:**
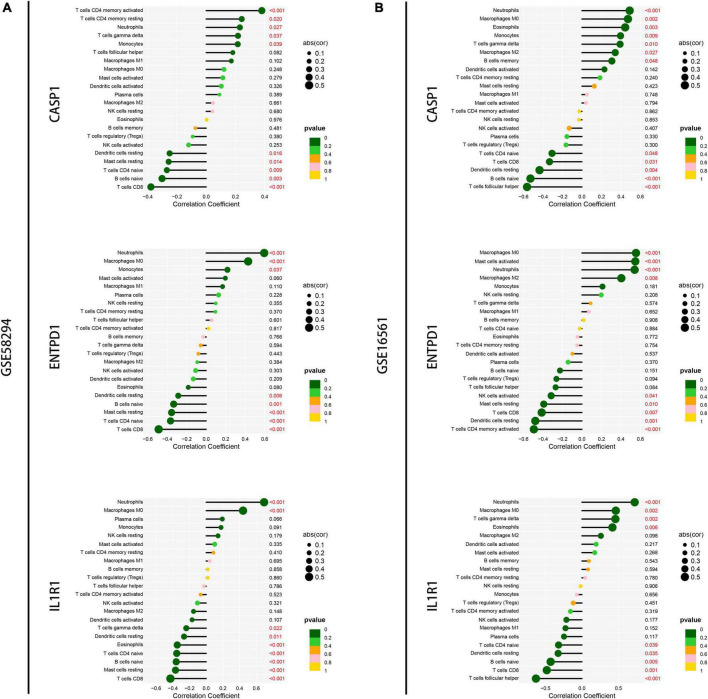
Correlation analysis of immune infiltration and signature genes expression. **(A)** Correlation of the top three signature genes expressions with immune infiltration was analyzed in the GSM58294 dataset. Those marked in red indicate statistically significant. **(B)** Correlation of the top three signature genes expressions with immune infiltration was analyzed in the GSM16561 dataset. Those marked in red indicate statistically significant.

**FIGURE 10:**
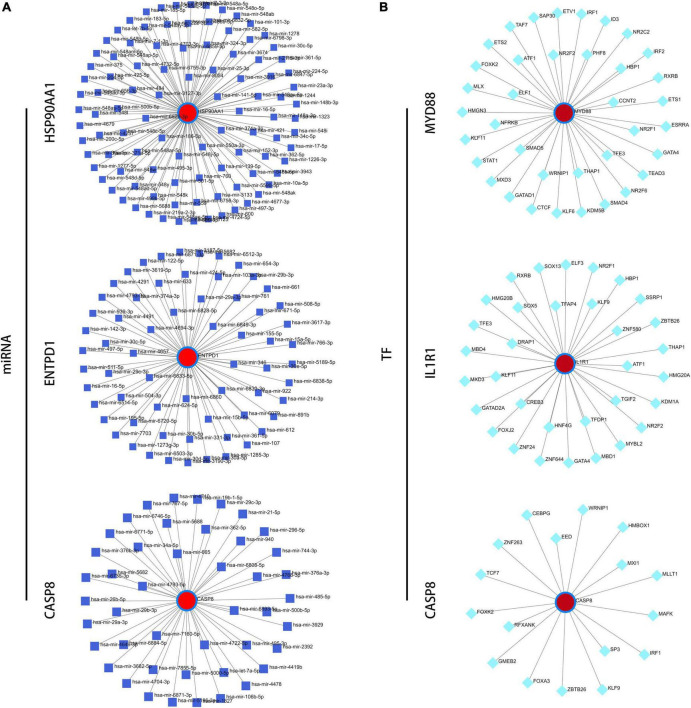
Construction of regulatory networks. **(A)** The gene-miRNA regulatory networks of the top three genes. **(B)** The gene-TF regulatory networks of the top three genes.

Next, we performed a correlation analysis between gene expression and immune cell infiltration levels for the first three diagnostic genes in the training set and validation set. The results showed that the expression of these genes was associated with the level of infiltration in multiple immune cells, which suggests that these key diagnostic genes are likely to be involved in immune regulation in the pathogenesis of IS (Figures 9A,B). In addition, we constructed the gene-miRNA and gene-TF regulatory networks: we show the networks of the top three genes in Figure 10. The results suggest that there are numerous miRNAs and TFs involved in the regulation of these diagnostic genes, which provides us with directions for subsequent therapeutic targeting of these genes.

## Discussion

Ischemic stroke, as a serious disease with a high incidence and mortality rate, often leads to lifelong disability in adults and places substantial stress and burdens on the patient’s family and society. For a long time, researchers have been working to improve the early preclinical diagnosis and treatment of IS. Cell death is divided into regulated cell death (RCD) and accidental cell death (ACD) (Galluzzi et al., 2018). RCD, such as apoptosis, necrosis, autophagy, ferroptosis, copper-induced cell death and immunogenic cell death, can be regulated by pharmacological or genetic interventions, which have been extensively studied in many diseases and have contributed to the development of many therapeutic approaches (Lai et al., 2018; Sun et al., 2018). However, few studies have examined the role of ICD in non-infectious, non-malignant diseases, such as stroke (Kroemer et al., 2022). In our study, multiple machine learning algorithms were used to explore the role of ICD-related genes in IS. For the first time, we analyzed ICD expression profiles, performed clustering analysis, analyzed immune infiltration, screened for prognostic signature genes, and built IS risk models.

We obtained 18 differentially expressed genes by differential analysis of samples from the IS patient group and normal control samples, and the coexpression analysis of these genes revealed many synergistic effects between them, especially HSP90AA1 and LY96 and ENTPD1 and IL1R1. Our clustering analysis showed that based on these differentially expressed genes, we could classify IS patients into two clusters. The immunoinfiltration profile of these two clusters was fully analyzed.

Neutrophils are among the first immune cells to be recruited to the ischemic brain, and it has been reported that increased levels of peripheral blood neutrophil-to-lymphocyte ratio (NLR) at the time of admission represent an independent risk factor for deterioration in neurological function and high rates of mortality (Iadecola et al., 2020). Mast cells contain granules with vasoactive agents and proteases that have been implicated in the destruction of the blood brain barrier (BBB) and extravasation of neutrophils in cerebral ischemia, and the deficiency of mast cells or pharmacological inhibition of mast cells exerts a neuroprotective effect (Strbian et al., 2006; Lindsberg et al., 2010). It has been reported that the secretion of protective remodeling factors by M2 macrophages can promote neuronal network recovery through tissue (including neuronal) and vascular remodeling (Kanazawa et al., 2017). In addition, increased monocytes might be related to IS volume and poor outcome, whereas suppression of the recruitment of monocytes significantly reduces post-IS brain oedema (Park et al., 2020; Qiu et al., 2021). In our study, Cluster B displayed significantly elevated levels of neutrophils and monocytes and lower levels of M2 macrophages and resting mast cells, which indicated that the patients in Cluster B might have poor prognosis. In addition, we could administer different immunotherapies according to the level of immune infiltration in these patients.

As machine learning research continues to progress, machine learning algorithms are being proven to better characterize the complex and unpredictable nature of human physiology, and the use of this technology in the medical field continues to produce exciting results (Heo et al., 2019). In our study, we filtered the signature genes by using LASSO and SVM algorithms, and nine signature genes were obtained by combining the results of both algorithms. The nine signature genes offer relatively good diagnostic value in both the training and validation sets, and in addition, a nomogram containing nine genes can combine nine signature genes to better diagnose the occurrence of IS.

CASP1 plays an important role in the classical pathway of pyroptosis, a cell death pathway involved in the pathology of acute cerebral ischemia, and studies have shown that inhibition of CASP1 activation in IS can rescue infarct volume, promote motor recovery, and improve behavioral outcomes in mouse stroke models (Li et al., 2020; Ma et al., 2020). Wong et al. (2019) found that IL-1R1 mediates the deleterious effects of IL-1 in ischemic stroke brain and that targeting cell-specific IL-1R1 in the brain may confer beneficial therapeutic effects for stroke and other cerebrovascular diseases. Clinical studies with large samples have shown that elevated caspase-8 levels are associated with an increased incidence of ischemic stroke (Muhammad et al., 2018). These studies involving these signature genes showed to some extent that the results of our screening are reliable. In addition, further analysis of these signature genes, including exploring their immune correlation and their interaction network with miRNAs, TFs, and other regulatory factors, could provide us with directions for subsequent targeting and immunotherapy of IS. In the future, we will continue to explore their potential mechanisms of action in IS through molecular biology experiments.

In conclusion, our study provides the first comprehensive analysis of the role of ICD-related genes in IS. In this study, we demonstrated consensus clustering analysis and machine learning analysis based on ICD-related genes and their roles in immune infiltration and diagnosis of IS. Our study may provide a valuable reference for further elucidation of the pathogenesis of IS and provide directions for drug screening, personalized therapy, and immunotherapy for IS.

## Data availability statement

The datasets presented in this study can be found in online repositories. The names of the repository/repositories and accession number(s) can be found in the article/Supplementary material.

## Author contributions

JC, QC, and SZ designed the research. JC, YH, and ZY downloaded and analyzed the data. ZY, YH, JC, LZ, LW, FY, and JY wrote the manuscript. All authors read and approved the final manuscript.

## Footnotes

1
http://www.ncbi.nlm.nih.gov/geo

2
http://www.networkanalyst.ca

## Supplementary material

Supplementary Figure 1 **(A,B)** Heatmap and boxplot showing the expression of the nine signature genes in the GSE16561 dataset.
