# Supplementary figures and images for: Identification of immunogenic cell death-related gene classification patterns and immune infiltration characterization in ischemic stroke based on machine learning
Source: Front Cell Neurosci. 2022 Dec 19;16:1094500. doi: 10.3389/fncel.2022.1094500 (PMC9806121; doi:10.3389/fncel.2022.1094500)

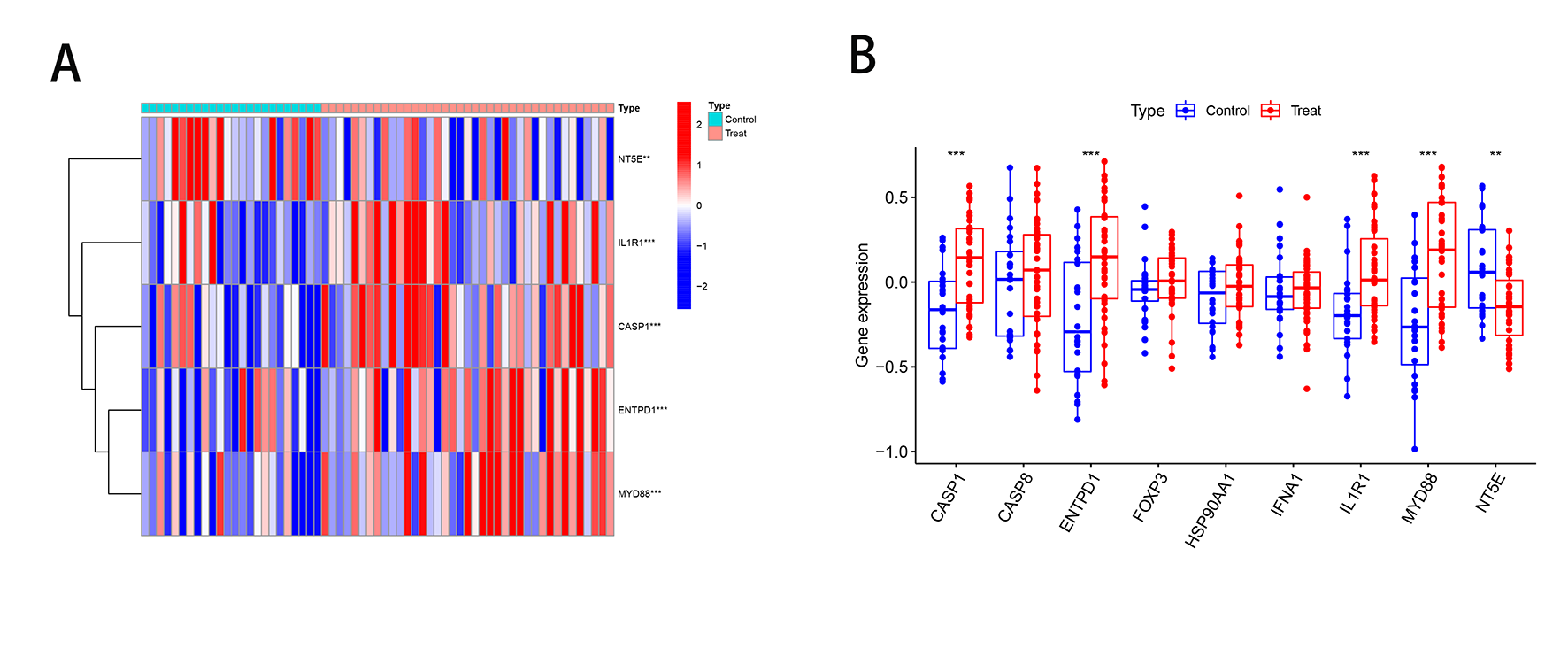

Supplement: Supplementary Figure 1 — (A,B) Heatmap and boxplot showing the expression of the nine signature genes in the GSE16561 dataset. [file Image_1.TIF]
